# Enhanced Bone Regeneration through Regulation of Mechanoresponsive FAK-ERK1/2 Signaling by ZINC40099027 during Distraction Osteogenesis

**DOI:** 10.7150/ijms.88298

**Published:** 2024-01-01

**Authors:** Shanyu Li, Hongxiao Wu, Feng Wang, Lingchi Kong, Yifan Yu, Rongtai Zuo, Haoyu Zhao, Jia Xu, Qinglin Kang

**Affiliations:** 1 Department of Orthopedics, Shanghai Sixth People's Hospital Affiliated to Shanghai Jiao Tong University School of Medicine, Shanghai, PR China.; 2 Department of Orthopedics, Dongying People's Hospital, Dongying, Shandong, PR China.

**Keywords:** Distraction osteogenesis, Focal adhesion kinase, ZINC40099027, Angiogenesis, Bone regeneration, Mechanical stimulation

## Abstract

**Background:** Focal adhesion kinase (FAK) is activated by mechanical stimulation and plays a vital role in distraction osteogenesis (DO), a well-established but lengthy procedure for repairing large bone defects. Both angiogenesis and osteogenesis contribute to bone regeneration during DO. However, the effects of ZINC40099027 (ZN27), a potent FAK activator, on angiogenesis, osteogenesis, and bone regeneration in DO remain unknown.

**Methods:** The angiogenic potential of human umbilical vein endothelial cells (HUVECs) was evaluated using transwell migration and tube formation assays. The osteogenic activity of bone marrow mesenchymal stem cells (BMSCs) was assessed using alkaline phosphatase (ALP) and alizarin red s (ARS) staining. Additionally, quantitative real-time polymerase chain reaction (qRT-PCR), western blot, and immunofluorescence staining were used to assay angiogenic markers, osteogenic markers, and FAK-extracellular signal-regulated kinase 1/2 (ERK1/2) signaling. *In vivo*, a rat tibia DO model was established to verify the effects of ZN27 on neovascularization and bone regeneration using radiological and histological analyses.

**Results:** ZN27 promoted the migration and angiogenesis of HUVECs. Additionally, ZN27 facilitated the osteogenic differentiation of BMSCs, as revealed by increased ALP activity, calcium deposition, and expression of osteogenesis-specific markers. The ERK1/2-specific inhibitor PD98059 significantly hindered the effects of ZN27, suggesting the participation of FAK-ERK1/2 signaling in ZN27-enhanced angiogenesis and osteogenesis. As indicated by improved radiological and histological features, ZN27 induced active angiogenesis within the distraction area and accelerated bone regeneration in a rat DO model.

**Conclusion:** Our results show that ZN27 targets FAK-ERK1/2 signaling to stimulate both angiogenesis and osteogenesis, and ZN27 accelerates bone regeneration in DO, suggesting the therapeutic potential of ZN27 for repairing large bone defects in the mechanobiological environment during DO.

## Introduction

Distraction osteogenesis (DO) is a well-established procedure for correcting critical-sized bone defects, nonunions, and skeletal deformities 1-3. Three phases occur sequentially during the DO procedure: latency, distraction, and consolidation. In the latency phase, a hematoma forms after osteotomy. In the distraction phase, the osteotomized bone segments are separated by gradual rhythmic distraction to a desired length. In the consolidation phase, newly formed bone gradually becomes mineralized until sufficient stability is achieved 4, 5. A major limitation of DO is the long-term consolidation, which leads to increased complications as well as psychological and economic burdens 6, 7. Therefore, it is of great interest to enhance bone regeneration and shorten treatment duration.

Mechanical forces play a crucial role in maintaining the structural stability and regenerative potential of bone, especially during DO 8, 9. Focal adhesion kinase (FAK), a non-receptor tyrosine kinase associated with integrins, is one of the key regulators in the transduction of mechanical signals 10, 11. Lawrence et al. observed FAK expression within the distraction gap in mandibles during DO, but not in bone defects created by acute separation, despite varying degrees of bone regeneration 12. Furthermore, FAK inhibition results in the disruption of bone regeneration during DO, and the expression of FAK is temporally parallel to distraction forces, with significant downregulation of FAK in the consolidation phase compared to gradual distraction 13, 14, making FAK an attractive target for enhancing bone regeneration. During DO, bone regeneration is a complex process, which includes multipotent cell differentiation, angiogenesis, and osteogenesis 15. As the key participants, endothelial cells and bone marrow mesenchymal stem cells (BMSCs) are reportedly regulated by FAK via FAK- extracellular signal-regulated kinase 1/2 (ERK1/2) signaling, which results in enhanced angiogenesis and osteogenesis, respectively 16, 17. These findings suggest that FAK may augment angiogenesis and osteogenesis in the mechanobiological environment during DO through the activation of the FAK-ERK1/2 signaling, thereby promoting bone regeneration.

Despite the development of FAK inhibitors for cancer therapies 18, specific FAK activators are lacking. Recently, a small molecule activator of FAK, ZINC40099027 (ZN27), was identified through ligand-based screening 19. ZN27 reportedly promotes gastrointestinal epithelial monolayer defect closure, and therefore accelerates mucosal ulcer healing 20, 21. Angiogenesis is essential for mucosal healing 22. Moreover, the biological activities of ZN27 occur through direct interaction with the kinase domain of FAK and subsequent activation of ERK1/2 23, 24, which promotes angiogenesis, implying the pro-angiogenic potential of ZN27. In addition, considering that the FAK-ERK1/2 signaling is critical for the osteogenic differentiation of BMSCs, we hypothesized that ZN27 treatment may enhance bone regeneration by facilitating both angiogenesis and osteogenesis via the FAK-ERK1/2 signaling.

In this study, we evaluated the effects of ZN27 on the angiogenic potential of human umbilical vein endothelial cells (HUVECs) and osteogenic differentiation of BMSCs. Additionally, the participation of the FAK-ERK1/2 signaling underlying the regulation of angiogenesis and osteogenesis by ZN27 was explored. Finally, the therapeutic potential of ZN27 to facilitate neovascularization and bone regeneration was verified in a rat DO model.

## Materials and methods

### Cell culture

HUVECs and endothelial cell medium (ECM) were obtained from ScienCell Corporation (Shanghai, China). For the culture of HUVECs, ECM supplemented with 5% (v/v) fetal bovine serum (FBS; ScienCell) and 1% (v/v) endothelial cell growth supplement (ScienCell) was used. BMSCs were isolated from 2-week-old male Sprague-Dawley (SD) rats by rinsing the femoral and tibial bone marrows. The isolated cells were cultured in Minimum Essential Medium Alpha basic (α-MEM; Gibco, Grand Island, NY, USA) containing 10% FBS (Gibco). After reaching 80-90% confluence, the cells were passaged. The cells between passage 2 and 4 were used for further experiments. Both HUVECs and BMSCs were cultured in a humidified atmosphere of 5% CO2 at 37 °C.

### Chemicals

ZN27 was obtained from Enamine (Monmouth Junction, NJ, USA), and applied at serial concentrations (0,10,100,500,1000 nM). PD98059, a selective inhibitor of ERK1/2, was obtained from MedChemExpress (Monmouth Junction, NJ, USA), and used at a concentration of 50 µM and 10 µM when incubated with HUVECs and BMSCs, respectively.

### Cell proliferation assay

BMSCs or HUVECs (2 × 10^3^/well) were seeded onto 96-well plates and incubated for 3 days. After 1 day, 2 days, and 3days of incubation, the cells were incubated with CCK-8 reagent (Dojindo, Kumamoto, Japan) for another 2 h. The optical density (OD) value at 450 nm was measured.

### Cell migration assay

HUVECs (2 × 10^4^/well) were seeded into the upper chamber of 8-µm pore-size, 24-well Transwell plates (Corning, NY, USA), and 200 μL serum-free ECM and 500 μL ECM complete medium were added into the upper and lower chambers, respectively. After 24 h, a cotton swab was used to remove unmigrated cells on the upper chamber surface. Migrated cells were fixed in 4% (w/v) paraformaldehyde (PFA; Servicebio, Wuhan, China), and then stained with 0.1% (w/v) crystal violet (Solarbio, Beijing, China). The migrated cells were counted under a light microscope (Nikon TE2000-E, Tokyo, Japan).

### Tube formation assay

HUVECs (2 × 10^4^/well) were seeded into 96-well plates precoated with Matrigel (Corning). After the cells were incubated in serum-free medium for 6 h, Calcein-AM (2 μg/ml) (Solarbio) was used to stain the living cells for 30 min, then the dye-containing medium was replaced by serum-free medium. Pseudo capillary networks were imaged using a fluorescence microscope (Leica DMi8, Wetzlar, Germany), then total tube length was measured using Image J software (NIH, Bethesda, MD, USA).

### Osteogenic differentiation induction and detection

BMSCs (3 × 10^4^/well) were seeded onto 24-well plates. One day after seeding, α-MEM complete medium was replaced by osteogenic differentiation induction medium (OIM; Cyagen, Guangzhou, China). The medium was replaced every 3 days. After osteogenic induction for 7 days, alkaline phosphatase (ALP) staining was conducted using ALP dye liquor (Beyotime, Shanghai, China). The staining was imaged under a light microscope, then the positive staining area was evaluated using Image J software. On day 14 of induction, alizarin red S (ARS; Cyagen) staining was employed to detect mineral deposition. The images of staining were observed using a light microscope. To quantify the staining results, 10% (w/v) cetylpyridinium chloride (Sigma-Aldrich, St. Louis, MO, USA) was used to elute the calcium deposition, then the absorbance at 570 nm was measured.

### Quantitative real-time polymerase chain reaction (qRT-PCR)

Total RNA of the cultured cells was extracted using an RNA Purification Kit (EZBioscience, Roseville, MN, USA). After measuring the concentration of RNA, Complementary DNA was synthesized using a Reverse Transcription Kit (EZBioscience) and used as template to perform qRT-PCR using SYBR Green Master Mix (EZBioscience). Primers were obtained from Sangon Biotech (Shanghai, China) (Table 1). Glyceraldehyde-3-phosphate dehydrogenase (GAPDH) served as internal control.

### Western blot

Total protein of the cultured cells was extracted using RIPA lysis buffer (Beyotime). A bicinchoninic acid kit (EpiZyme, Shanghai, China) was used to detect the protein concentration. Proteins were separated by 10% (w/v) SDS-PAGE and then blotted to polyvinylidene difluoride membrane (Bio-Rad, Hercules, CA, USA). After that, the membrane was blocked with 5% (w/v) bovine serum albumin (BSA) for 1 h at room temperature, treated with primary antibodies overnight at 4°C, and then incubated with horseradish peroxidase (HRP)-conjugated secondary antibodies (1:2000, ab6721, Abcam, Cambridge, UK) for 1 h at room temperature. The target bands on the membrane were visualized using ECL reagent (EpiZyme).

Primary antibodies against FAK (1:1000, #3285, Cell Signaling Technology, Danvers, MA, USA), p-FAK (1:1000, ab81298, Abcam), ERK1/2 (1:1000, #4695, Cell Signaling Technology), p-ERK1/2 (1:1000, #4370, Cell Signaling Technology), RUNX2 (1:1000, #12556, Cell Signaling Technology), p-RUNX2 (1:2000, #AF7379, Affinity Biosciences, Cincinnati, OH, USA), and GAPDH (1:2000, #5174, Cell Signaling Technology) were used in this study.

### Animal surgery and intervention

Male SD rats were housed with free access to food and drinking water in a 12-hour light/dark cycle at room temperature (23 ± 1°C). All animal experiments followed the National Institutes of Health Guide for the Care and Use of Laboratory Animals and were approved by the Animal Welfare Ethics Committee of Shanghai Sixth People's Hospital Affiliated to Shanghai Jiao Tong University School of Medicine (No. DWLL2023-0233).

Twenty-four 12-week-old male SD rats were used for experiments and randomly assigned to 2 groups (n = 12 per group): (1) control group, (2) ZN27 group. The surgical procedures to establish a rat DO model were described as followed. The rats were anesthetized with intraperitoneal injection of 2% pentobarbital (40 mg/kg). After exposure of surgical area, a monolateral external fixator (Xinzhong Company, Tianjin, China) was mounted to the right tibia, which was transversely osteotomized at the midshaft. Thereafter, the surgical incisions were sutured. Three phases occurred sequentially after the surgery: latency phase for 5 days, distraction phase for 10 days (0.25 mm/12 h), and consolidation phase for 4 weeks. PBS and ZN27 (900 µg/kg/6h) at the same dosage were intraperitoneally injected to the rats in the control and ZN27 group respectively during the consolidation phase. After consolidation for 2 and 4 weeks, the rats were euthanized with overdose injection of 2% pentobarbital (200 mg/kg) (n = 6 per group at each time point). Tibia specimens were then harvested and subject to micro-CT scanning (n = 6 per group at each time point), histological and immunohistochemical staining (n = 3 per group at each time point), and immunofluorescence staining (n = 3 per group at each time point).

### Micro-CT analysis

Tibia specimens were subject to Micro-CT scanning (Skyscan 1076; Bruker, Kontich, Belgium) (18-μm thickness per slide). The scanned images were used for three-dimensional reconstruction for the regenerated callus using CTvox software (Bruker) and for quantification of bone volume/tissue volume (BV/TV) of the regenerated area (5 mm within the distraction region) for each specimen using CTan software (Bruker).

### Histology and immunohistochemistry

After fixation with 4% PFA for 24 h and decalcification with 10% (w/v) ethylenediaminetetraacetic acid (EDTA) for 3 weeks, tibia specimens were dehydrated with gradient ethanol, embedded in paraffin, and cut into sections (5 µm thick). For all staining, the sections that met the following criteria were used for analysis: consistent thickness, transparent appearance, complete tissue structures, no ruptures or folds, and clear contrast. Hematoxylin and eosin (H&E), Masson's trichrome, and Picrosirius red (PSR) staining were performed in the sections. For immunohistochemical staining, the sections first underwent incubation with primary antibodies overnight at 4°C, followed by incubation with anti-rabbit (1:200, ab6721, Abcam) or anti-mouse (1:200, ab6728, Abcam) HRP-conjugated secondary antibodies for 1 h at room temperature. An HRP-streptavidin system (Dako, Glostrup, Denmark) was employed to detect the positive area, followed by counterstaining with hematoxylin.

Primary antibodies against COL2 (1:100, #AF0135, Affinity Biosciences), OCN (1:200, ab198228, Abcam), p-ERK1/2 (1:200, #4370, Cell Signaling Technology), p-RUNX2 (1:100, #AF7379, Affinity Biosciences), and VEGFA (1:200, ab1316, Abcam) were used in this study.

### Immunofluorescence staining

The samples from BMSCs first underwent fixation in 4% PFA for 15min and permeabilization using 0.2% (v/v) Triton X-100 for 10 min, followed by blocking with 5% BSA for 30 min. After that, the samples were treated overnight at 4°C with primary antibodies against p-RUNX2 (1:300, #AF7379, Affinity Biosciences), and then incubated with Alexa Fluor 488-conjugated secondary antibodies (1:200, ab150077, Abcam) for 1 h at room temperature. Nuclei in the cells were counterstained with 4',6-diamidino-2-phenylindole (DAPI) for 5 min, and the fluorescence images were observed under a fluorescence microscope.

Tibia specimens underwent fixation in 4% PFA overnight, decalcification in 18% EDTA for 7 days at 4 °C, and dehydration with 30% (w/v) sucrose. After that, the specimens were embedded in optimal cutting temperature compound and cut into sections (10 µm thick). Immunofluorescence staining of CD31 and endomucin (EMCN) was performed in samples from tibia sections using primary antibodies against CD31 (1:100, ab64543, Abcam) and EMCN (1:100, sc-65495, Santa Cruz Biotechnology, Dallas, TX, USA) overnight at 4 °C. After incubation with Cy3 (1:200, ab97035, Abcam) and Alexa Fluor 488 (1:200, ab150157, Abcam)-conjugated secondary antibodies and counterstaining with DAPI, the fluorescence images were observed under a fluorescence microscope.

### Statistical analysis

All data are presented as mean ± standard deviation. GraphPad Prism 9 software (GraphPad Prism, La Jolla, CA, USA) was applied for statistical analysis. Student's t test was used for comparison of two groups, and one-way or two-way ANOVA followed by Tukey's or Sidak's multiple comparisons test was used for comparison among multiple groups. A two-tailed p < 0.05 was accepted as statistically significant.

## Results

### ZN27 enhanced angiogenesis through the FAK-ERK1/2 signaling

**Figure 1:**
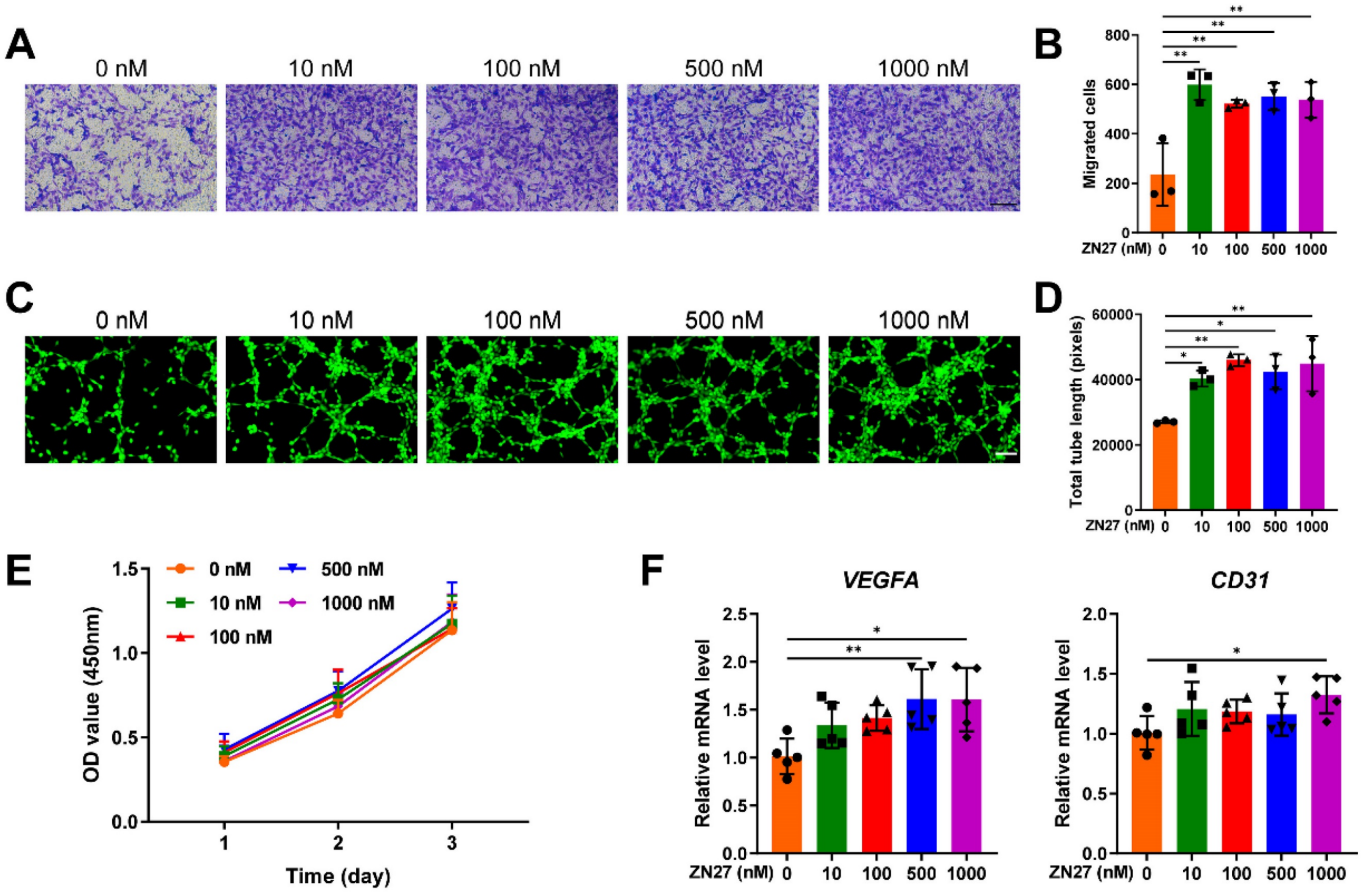
Effects of ZN27 on angiogenesis in HUVECs. **A** The migration capacity of HUVECs after ZN27 treatment was detected using Transwell assay, and the migrated cells were stained with crystal violet. Scale bar: 200 µm. **B** Quantitative analysis of the migrated cells. n = 3 per group. **C** The angiogenic ability of HUVECs treated with ZN27 was evaluated using tube formation assay, and the living cells that formed tube-like structures were stained with calcein. Scale bar: 100 µm. **D** Quantitative analysis of the tube-like structures. n = 3 per group.** E** Effects of ZN27 on HUVEC proliferation were analyzed using CCK-8 assay. n = 5 per group. **F** qRT-PCR analysis showed the relative expression levels of *VEGFA* and *CD31* in HUVECs treated with ZN27 for 1 day. n = 5 per group. Abbreviations: ZN27, ZINC40099027; VEGFA, vascular endothelial growth factor A; CD31, platelet endothelial cell adhesion molecule-1; HUVECs, human umbilical vein endothelial cells. Data are presented as the mean ± standard deviation. For **B**, **D**, and **F**, one-way ANOVA with Tukey's multiple comparisons test was employed. For **E**, two-way ANOVA with Tukey's multiple comparisons test was used. *p < 0.05; **p < 0.01.

**Figure 2:**
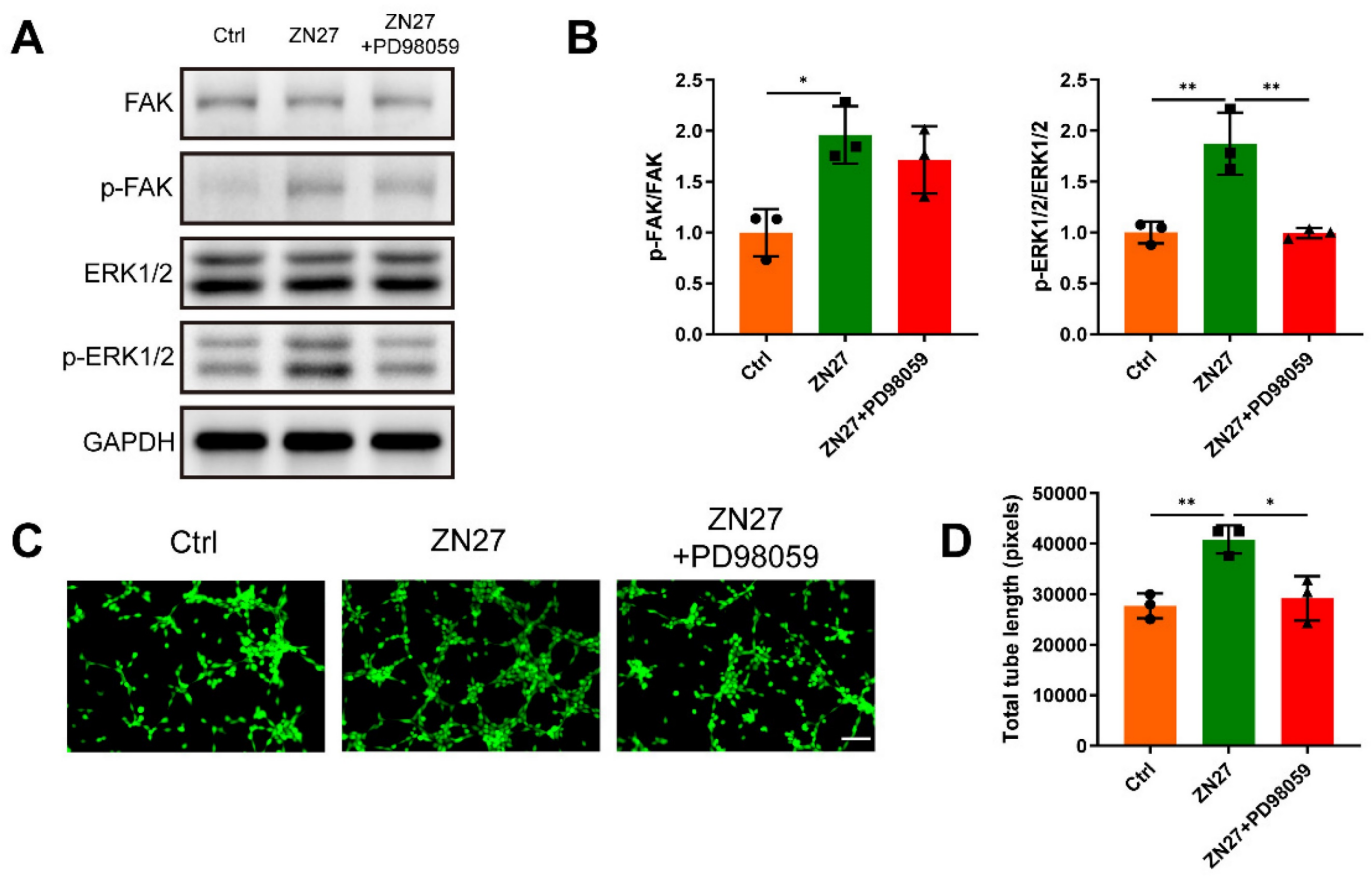
ZN27 activated the FAK-ERK1/2 signaling to enhance angiogenesis in HUVECs. **A, B** Western blot was used to identify the expression of FAK, p-FAK, ERK1/2, and p-ERK1/2 at the protein level in HUVECs treated with ZN27 and PD98059 for 1 day, followed by quantitative analysis. n = 3 per group. **C** The angiogenic ability of HUVECs treated with ZN27 and PD98059 was evaluated using tube formation assay, and the living cells that formed tube-like structures were stained with calcein. Scale bar: 100 µm. **D** Quantitative analysis of the tube-like structures. n = 3 per group. PD98059: ERK1/2 inhibitor. Abbreviations: ZN27, ZINC40099027; FAK, focal adhesion kinase; ERK1/2, extracellular signal-regulated kinase 1/2; GAPDH, glyceraldehyde-3-phosphate dehydrogenase; HUVECs, human umbilical vein endothelial cells. Data are presented as the mean ± standard deviation. For **B** and **D**, one-way ANOVA with Tukey's multiple comparisons test was employed. *p < 0.05; **p < 0.01.

HUVECs were incubated with serial concentrations of ZN27 for angiogenic experiments. Transwell assay confirmed that ZN27 facilitated the migration capacity of HUVECs (Fig. 1A, B). The *in vitro* angiogenic capacity of HUVECs was evaluated using Matrigel tube formation assay. ZN27 led to improved angiogenesis, as indicated by increased total tube length (Fig. 1C, D). As detected by CCK-8 assay, ZN27 showed no significant cytotoxicity and did not promote HUVEC proliferation (Fig. 1E). In addition, the expression of angiogenic markers was evaluated using qRT-PCR, which showed that ZN27 augmented the expression of *VEGFA* and *CD31* (Fig. 1F). Regarding the underlying mechanisms of ZN27 promoting angiogenesis, western blot revealed that ZN27 (1000 nM) activated FAK and downstream signaling molecule ERK1/2, based on the elevated phosphorylation levels of FAK and ERK1/2 (Fig. 2A, B). Meanwhile, the activation of ERK1/2 was significantly hindered by the ERK1/2-specific inhibitor PD98059 (Fig. 2A, B). Moreover, the *in vitro* angiogenesis enhanced by ZN27 (1000 nM) was attenuated by PD98059 (Fig. 2C, D). These results indicate that ZN27 promotes the angiogenic capacity of HUVECs, which is attributed to the activation of the FAK-ERK/1/2 signaling.

### ZN27 promoted osteogenic differentiation of BMSCs

**Figure 3:**
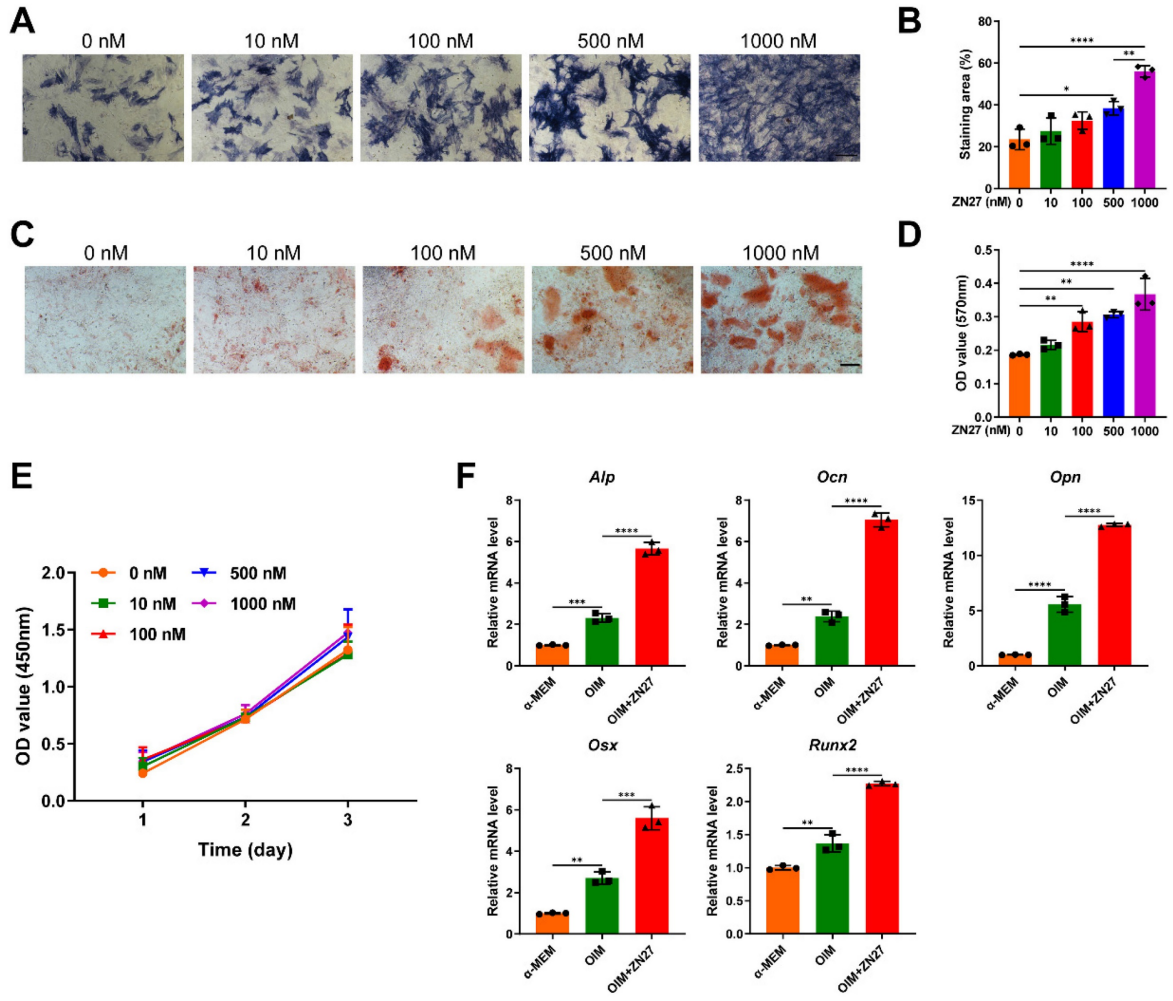
ZN27 enhanced osteogenic differentiation of BMSCs. **A, B** Osteogenic activity of BMSCs treated with OIM and serial concentrations of ZN27 for 7 days was evaluated using ALP staining, followed by quantitative analysis. Scale bar: 200 µm. n = 3 per group.** C, D** Calcium deposition of BMSCs was detected using ARS staining after 14 days of treatment with OIM and serial concentrations of ZN27, followed by quantitative analysis. Scale bar: 100 µm. n = 3 per group. **E** Effects of ZN27 on BMSC proliferation were analyzed using CCK-8 assay. n = 5 per group. **F** The relative expression levels of osteogenesis-specific genes in BMSCs treated with OIM and ZN27 (1000 nM) for 5 days were assessed using qRT-PCR. n = 3 per group. PD98059: ERK1/2 inhibitor. Abbreviations: ZN27, ZINC40099027; ALP, alkaline phosphatase; OCN, osteocalcin; OPN, osteopontin; OSX, osterix; RUNX2, runt-related transcription factor 2; α-MEM, minimum essential medium alpha basic; OIM, osteogenic differentiation induction medium; BMSCs, bone marrow mesenchymal stem cells; ARS, alizarin red S. Data are presented as the mean ± standard deviation. For **B**, **D**, and **F**, one-way ANOVA with Tukey's multiple comparisons test was used. For **E**, two-way ANOVA with Tukey's multiple comparisons test was employed. *p < 0.05; **p < 0.01; ***p < 0.001; ****p < 0.0001.

The osteogenic capacity of BMSCs after ZN27 treatment was assessed using ALP and ARS staining on day 7 and 14 of osteogenic induction, respectively. The staining results showed that ZN27 (0-1000 nM) significantly enhanced ALP activity in a dose-dependent manner, with maximal activity observed at a concentration of 1000 nM (Fig. 3A, B). Calcium deposition detected by ARS staining exhibited similar trends (Fig. 3C, D). As shown in Fig. 3E, ZN27 had no significant cytotoxic or stimulatory effects on BMSC proliferation. Therefore, ZN27 at a concentration of 1000 nM was employed in the present study for further experiments. Using qRT-PCR, we observed that the expression of osteogenesis-specific genes (*Alp*, *Ocn*, *Opn*, *Osx*, and *Runx2*) was induced by OIM and further enhanced by ZN27 treatment (Fig. 3F).

### ZN27 mediated FAK-ERK1/2-RUNX2 signaling activation to enhance osteogenesis

**Figure 4:**
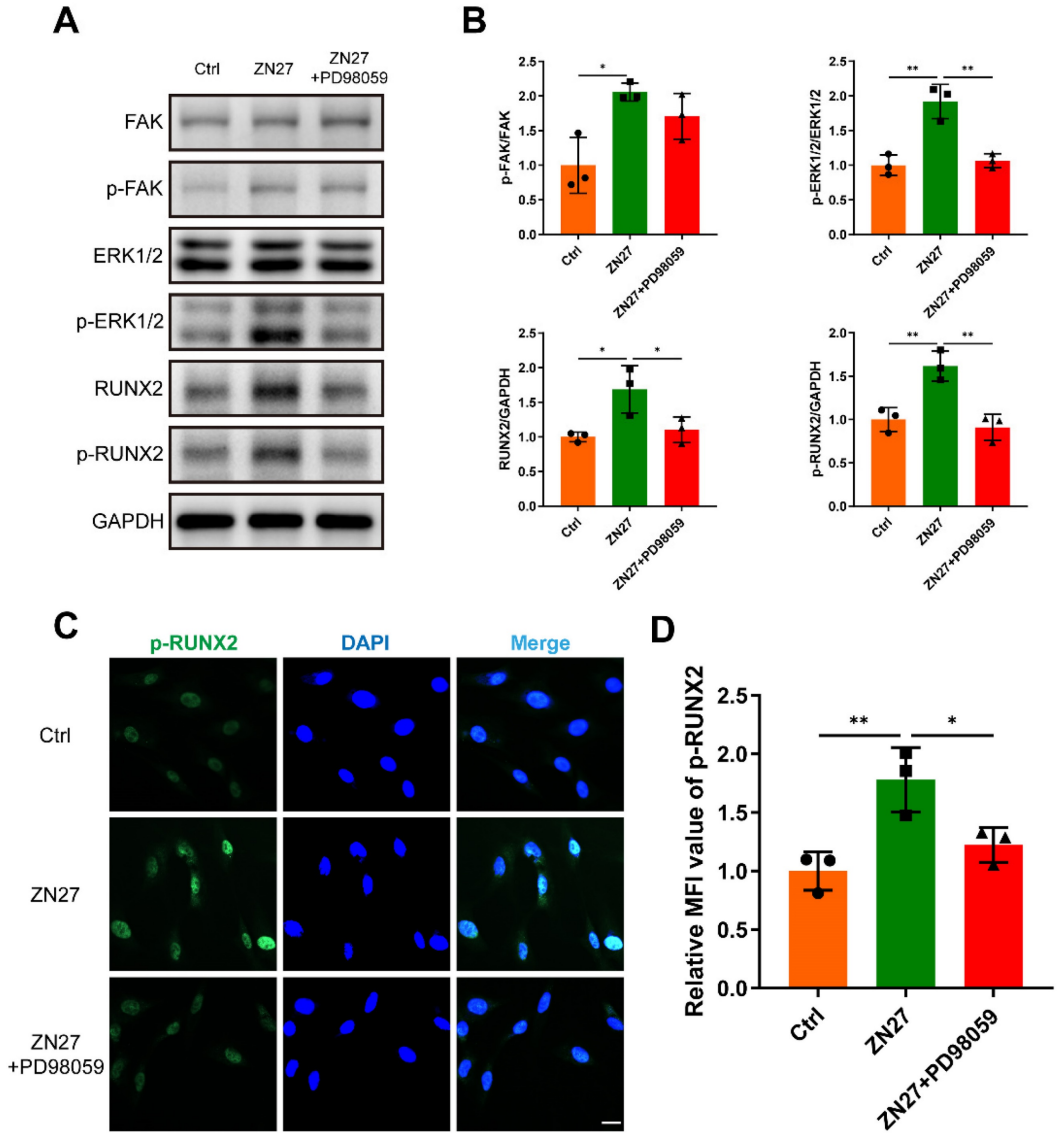
ZN27 activated the FAK-ERK1/2-RUNX2 signaling in BMSCs. **A, B** Western blot results of FAK, p-FAK, ERK1/2, p-ERK1/2, RUNX2, and p-RUNX2 in BMSCs treated with OIM, OIM+ZN27, and OIM+ZN27+PD98059 for 5 days, followed by quantitative analysis. n = 3 per group. **C, D** Immunofluorescence staining of p-RUNX2 in BMSCs treated with OIM, OIM+ZN27, and OIM+ZN27+PD98059, followed by quantitative analysis. Scale bar: 20 µm. n = 3 per group. PD98059: ERK1/2 inhibitor. Abbreviations: ZN27, ZINC40099027; FAK, focal adhesion kinase; ERK1/2, extracellular signal-regulated kinase 1/2; RUNX2, runt-related transcription factor 2; GAPDH, glyceraldehyde-3-phosphate dehydrogenase; BMSCs, bone marrow mesenchymal stem cells; OIM, osteogenic differentiation induction medium. Data are presented as the mean ± standard deviation. For **B** and **D**, one-way ANOVA with Tukey's multiple comparisons test was used. *p < 0.05; **p < 0.01.

**Figure 5:**
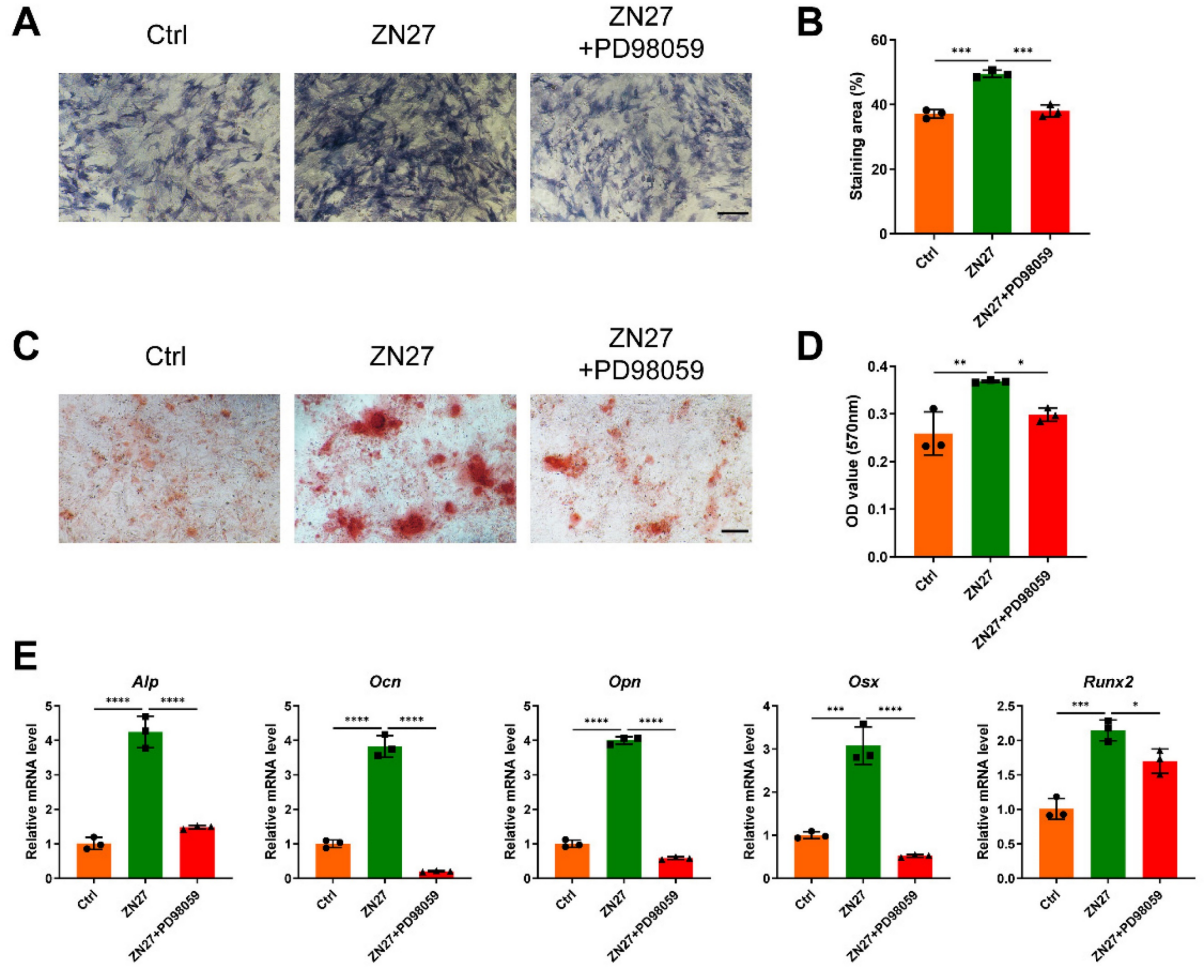
PD98059 inhibited the effects of ZN27 on osteogenic differentiation of BMSCs. **A, B** Osteogenic activity of BMSCs treated with OIM, OIM+ZN27, and OIM+ZN27+PD98059 for 7 days was assessed using ALP staining, followed by quantitative analysis. Scale bar: 200 µm. n = 3 per group.** C, D** Calcium deposition of BMSCs was detected using ARS staining after 14 days of treatment with OIM, OIM+ZN27, and OIM+ZN27+PD98059, followed by quantitative analysis. Scale bar: 100 µm. n = 3 per group. **E** The relative expression levels of osteogenesis-specific genes in BMSCs treated with OIM, OIM+ZN27, and OIM+ZN27+PD98059 for 5 days were detected using qRT-PCR. n = 3 per group. PD98059: ERK1/2 inhibitor. Abbreviations: ZN27, ZINC40099027; ALP, alkaline phosphatase; OCN, osteocalcin; OPN, osteopontin; OSX, osterix; RUNX2, runt-related transcription factor 2; BMSCs, bone marrow mesenchymal stem cells; OIM, osteogenic differentiation induction medium; ARS, alizarin red S. Data are presented as the mean ± standard deviation. For **B**, **D**, and **E**, one-way ANOVA with Tukey's multiple comparisons test was employed. *p < 0.05; **p < 0.01; ***p < 0.001; ****p < 0.0001.

Western blot suggested that ZN27 augmented the phosphorylation of FAK and ERK1/2 (Fig. 4A, B). In addition, RUNX2, a key transcription factor in osteogenesis and downstream of the FAK-ERK1/2 signaling, was upregulated at both the total and phosphorylated levels after ZN27 treatment (Fig. 4A, B). Simultaneously, the activation of ERK1/2 and RUNX2 was significantly attenuated by PD98059 (Fig. 4A, B). To further confirm this effect, we evaluated the levels of p-RUNX2 using immunofluorescence staining. Consistently, the mean fluorescence intensity (MFI) of p-RUNX2, initially elevated by ZN27, was compromised when BMSCs were co-cultured with PD98059 (Fig. 4C, D). Thereafter, we evaluated how the inhibition of ERK1/2 by PD98059 affected the osteogenic potential of BMSCs treated with ZN27 under osteogenic induction. As expected, ALP and ARS staining revealed lower ALP activity and calcium deposition after exposure to ZN27 and PD98059 compared to exposure to ZN27 alone (Fig. 5A-D). Similar inhibitory effects on the expression of osteogenesis-specific genes were observed (Fig. 5E). These results suggest the participation of the FAK-ERK1/2-RUNX2 signaling in ZN27-enhanced osteogenesis in BMSCs.

### ZN27 accelerated bone regeneration during distraction osteogenesis

**Figure 6:**
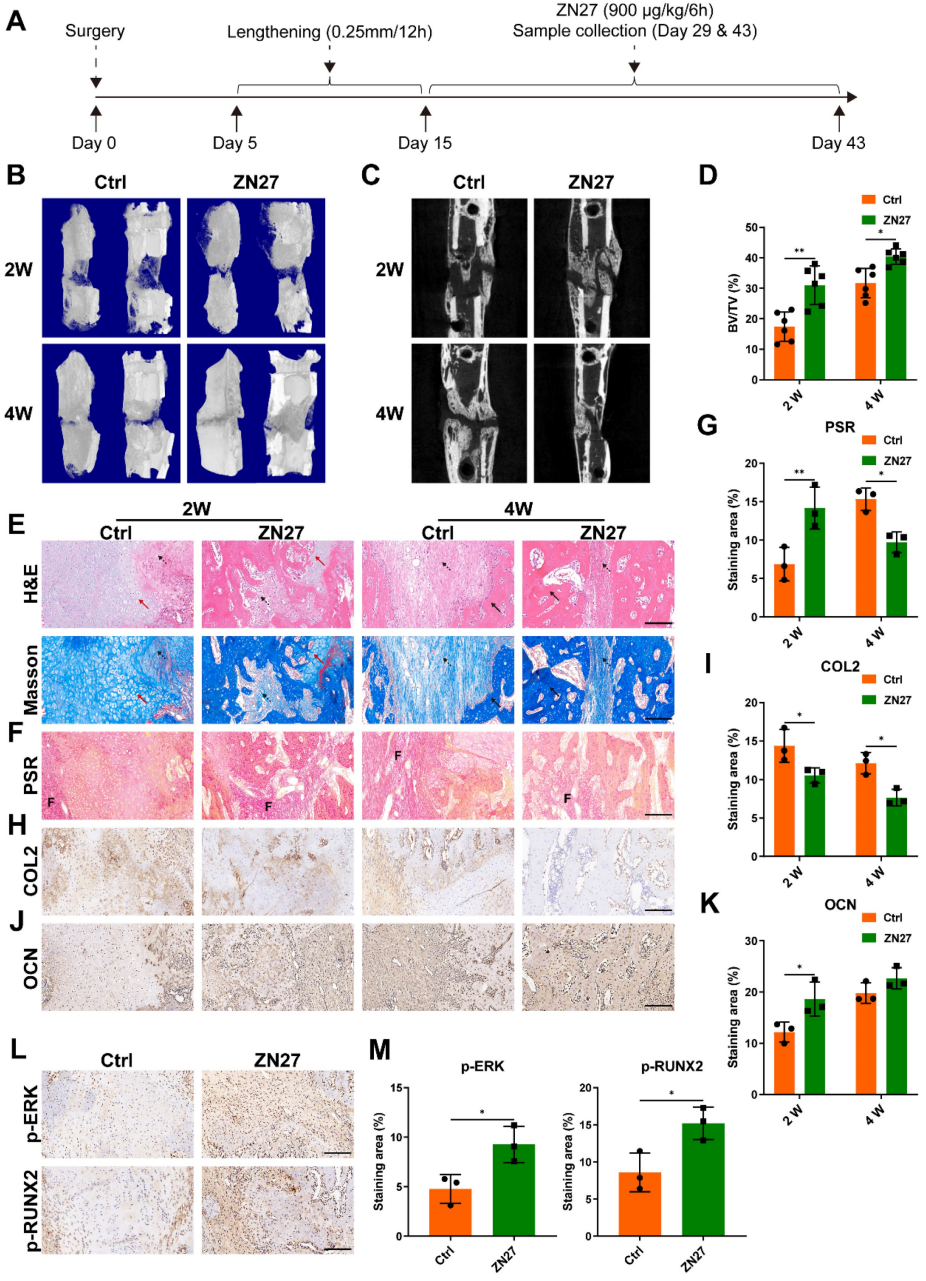
ZN27 promoted bone regeneration during distraction osteogenesis in rats. **A** Workflow of animal experiments. **B, C** Three-dimensional reconstructions (**B**) and longitudinal images (**C**) of micro-CT data of tibial distraction regenerates after consolidation for 2 and 4 weeks. For the three-dimensional reconstructions in each group, the left and right images were respectively the outside and inside views of the tibial distraction regenerates. **D** Quantitative analysis of BV/TV of the tibial distraction regenerates. n = 6 per group. **E** Representative images of H&E and Masson's trichrome staining in the central interzone of the tibial distraction regenerates. Red arrows, cartilaginous tissue. Dotted arrows, fibrous tissue. Black arrows, trabecular bone. Scale bar: 200 µm. **F, G** The fibrous tissue was stained with PSR (labeled with “F”), followed by quantitative analysis. Scale bar: 200 µm. n = 3 per group.** H, I** Immunohistochemical staining of chondrogenic marker COL2, followed by quantitative analysis. Scale bar: 200 µm. n = 3 per group.** J, K** Immunohistochemical staining of osteogenic marker OCN, followed by quantitative analysis. Scale bar: 200 µm. n = 3 per group.** L, M** Immunohistochemical staining of p-ERK and p-RUNX2 after consolidation for 2 weeks, followed by quantitative analysis. Scale bar: 200 µm. n = 3 per group. Abbreviations: ZN27, ZINC40099027; BV/TV, bone volume/tissue volume; H&E, hematoxylin-eosin; PSR, picrosirius red; COL2, collagen type II; OCN, osteocalcin; ERK1/2, extracellular signal-regulated kinase 1/2; RUNX2, runt-related transcription factor 2. Data are presented as the mean ± standard deviation. For **D**, **G**, **I**, and **K**, two-way ANOVA with Sidak's multiple comparisons test was used. For **M**, unpaired t test was employed. *p < 0.05; **p < 0.01.

Fig. 6A illustrated the workflow of animal experiments. In the experiment process, no rat died or underwent severe complications. During the consolidation phase, callus growth was enhanced by ZN27 administration, as revealed by the three-dimensional reconstructions and longitudinal images of tibial distraction regenerates (Fig. 6B, C). Similarly, BV/TV of ZN27-treated rats was elevated compared to that of untreated rats (Fig. 6D). H&E and Masson's trichrome staining of the regenerated bone revealed a central interzone with newly formed cartilaginous tissue, fibrous tissue, and trabecular bone (Fig. 6E). With the development of mineralization process, the cartilaginous and fibrous tissue were eventually replaced by trabecular bone. We performed picrosirius red (PSR) staining and immunohistochemical staining of collagen type II (COL2) to evaluate the fibrous and cartilaginous tissue, respectively. The staining results showed that less fibrous and cartilaginous tissue were observed in the ZN27 group than in the control group after consolidation for 4 weeks (Fig. 6F-I), suggesting that ZN27 accelerated the mineralization process. In addition, the immunohistochemical staining results showed higher expression and wider distribution of OCN in sections from the ZN27 group compared to those from the control group after consolidation for 2 weeks (Fig. 6J, K), which indicated more active osteogenesis in ZN27-treated rats at this time point. Thereafter, OCN expression in sections of untreated rats gradually increased (Fig. 6J, K), which demonstrated an active osteogenic process in untreated rats, although 2 weeks later than that observed in ZN27-treated rats. The immunohistochemical analysis also revealed elevated phosphorylation levels of ERK and RUNX2 in the ZN27 group than in the control group (Fig. 6L, M), indicating that FAK-ERK1/2-RUNX2 signaling participated in ZN27-enhanced bone regeneration during DO.

### ZN27 facilitated neovascularization of distraction regenerates

**Figure 7:**
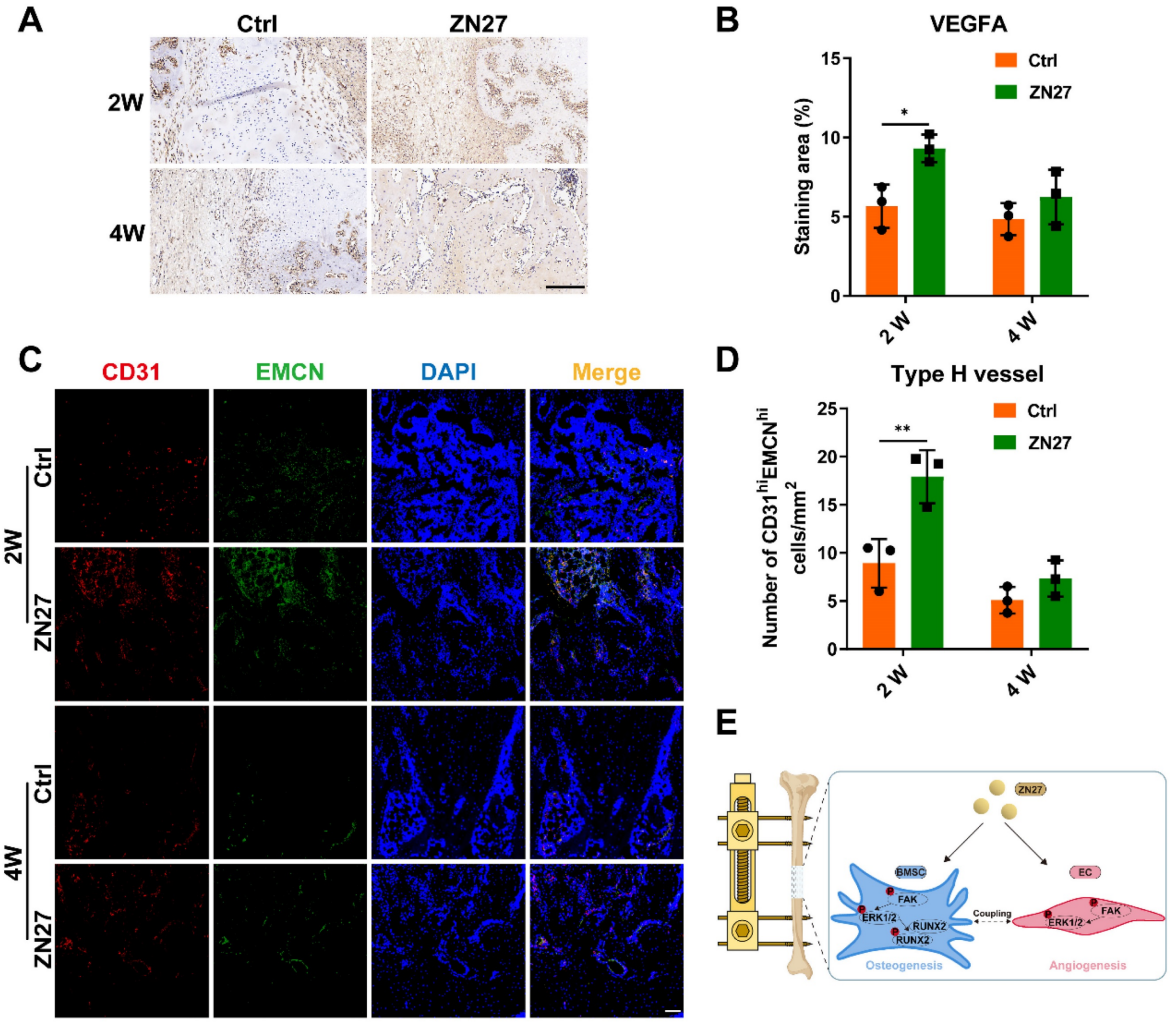
ZN27 improved neovascularization of distraction regenerates. **A, B** VEGFA expression after consolidation for 2 and 4 weeks was detected using immunohistochemical staining, followed by quantitative analysis. Scale bar: 200 µm. n = 3 per group. **C, D** Type H vessels were labeled using immunofluorescence staining of CD31 and EMCN, followed by quantitative analysis of CD31 and EMCN-positive cells. Scale bar: 100 µm. n = 3 per group. **E** Schematic diagram of ZN27-induced activation of FAK-ERK1/2 signaling promoting osteogenesis and angiogenesis during DO. Abbreviations: ZN27, ZINC40099027; VEGFA, vascular endothelial growth factor A; CD31, platelet endothelial cell adhesion molecule-1; EMCN, endomucin; BMSCs, bone marrow mesenchymal stem cells; ECs, endothelial cells; FAK, focal adhesion kinase; ERK1/2, extracellular signal-regulated kinase 1/2; RUNX2, runt-related transcription factor 2; DO, distraction osteogenesis. Data are presented as the mean ± standard deviation. For **B** and **D**, two-way ANOVA with Sidak's multiple comparisons test was used. *p < 0.05; **p < 0.01.

Regarding neovascularization, immunohistochemical staining for VEGFA was performed to observe angiogenic activities, and immunofluorescence staining of CD31 and EMCN was used to evaluate type H vessels. More prominent angiogenic activities and type H vessels were distributed within the distraction area in ZN27-treated rats than in untreated rats after consolidation for 2 weeks (Fig. 7A-D), indicating improved angiogenesis in the ZN27 group, although the angiogenic process became less active as mature trabecular bone increased after consolidation for 4 weeks.

## Discussion

Herein, we demonstrated that ZN27 promoted the angiogenic capacity of HUVECs and osteogenic differentiation of BMSCs *in vitro*, and the enhanced effects were attributed, at least in part, to ZN27-induced activation of the FAK-ERK1/2 signaling. In addition, ZN27 accelerated bone regeneration and promoted neovascularization in a rat DO model (Fig. 7E).

During the dynamic DO process, mechanical stimuli induced by tension stress lay the foundation for massive regenerative potential. A complex regulatory network is responsible for translating mechanical stimuli into intracellular biochemical responses for bone regeneration 25, 26. Recent studies have shown that FAK is activated by tension stress and elicits an endogenous biological reaction that induces new bone growth in the mandible or tibia during DO 13, 14, indicating the pivotal role of FAK signaling in tension stress-induced bone regeneration. Notably, ZN27 has been reported as a potent FAK activator 19, we therefore hypothesized that ZN27 promoted bone regeneration during DO via the FAK signaling. Although ZN27 has been shown to activate the FAK signaling in gastrointestinal mucosal ulcer repair 20, 21, to the best of our knowledge, this is the first study to apply ZN27 in a DO model.

Bone regeneration during DO relies on robust angiogenesis 27, 28. Enhancement of angiogenesis through various biological factors has been reported to facilitate bone regeneration in DO 4, 29-31, whereas impaired angiogenesis due to age or radiation exhibits the opposite effect 32, 33. Angiogenesis is a multi-step process, which includes endothelial cell activation, migration, and tube formation 34. As a key regulator of endothelial cell function 16, 35, 36, FAK plays an important role in angiogenesis by activating the FAK-ERK1/2 signaling 16, 37. In our study, ZN27 treatment promoted HUVEC migration and tube formation, as well as the expression of angiogenic markers *VEGFA* and *CD31*, with the participation of the FAK-ERK1/2 signaling *in vitro*. Notably, the proliferation of HUVECs was not affected by ZN27 treatment. This finding indicates that ZN27 promotes angiogenesis without stimulating proliferation, which is consistent with the results of previous studies 20, 21. Moreover, histological analysis showed that VEGFA expression and vascular growth were elevated within the distraction regenerates after ZN27 administration. Consistent with our *in vivo* results, a recent study has reported that calcitonin gene-related peptide promotes the angiogenic capacity of endothelial cells via FAK-induced VEGFA production within the distraction gap 38. As some angiogenic factors, including VEGFA, exhibit maximal upregulation in the distraction phase followed by downregulation during the consolidation phase 15, 27, our results and those of this study emphasize the crucial role of FAK in restoring the expression of VEGFA and therefore promoting angiogenesis in the DO model. In addition, previous studies have demonstrated that FAK kinase activity and nuclear translocation are critical for VEGF receptor 2 (VEGFR2) expression, with direct interaction between nuclear FAK and VEGFR2 promoter after VEGFA treatment in endothelial cells 39, 40, indicating that FAK activated by ZN27 during DO could facilitate angiogenesis through its synergistic effects on VEGFA/VEGFR2 expression; however, the interaction between nuclear FAK and VEGFR2 promoter induced by ZN27 in endothelial cells remains to be further investigated. Nonetheless, as illustrated by our data, ZN27 has therapeutic potential for facilitating angiogenesis during DO.

BMSCs are capable of differentiating into chondrogenic, adipogenic, or osteogenic lineages 41, and therefore provide a valuable source for bone regeneration 42. In human BMSCs, FAK phosphorylated by integrin α5 activates ERK1/2, upregulating RUNX2 expression, and leading to increased osteogenic differentiation 43, which indicates the pivotal role of FAK-ERK1/2-RUNX2 signaling in osteogenesis 10. Inspired by the potent FAK activation potential of ZN27, we evaluated the effects of ZN27 on BMSC osteogenic differentiation *in vitro* and bone formation *in vivo*. In our study, ZN27 promoted the osteogenic capacity of BMSCs and the FAK-ERK1/2-RUNX2 signaling was activated by ZN27 in BMSCs. Meanwhile, the enhanced signal transduction and pro-osteogenic effects induced by ZN27 were markedly weakened after exposure to the ERK1/2 selective inhibitor PD98059, suggesting that ZN27 facilitates BMSC osteogenic differentiation via the FAK-ERK1/2-RUNX2 signaling. Moreover, micro-CT and histological analyses revealed that ZN27 significantly accelerated callus formation and enhanced osteogenic activities within the distraction regenerates. It has been reported that activated FAK signaling in mesenchymal stem cells (MSCs) enhances angiogenesis during coupling with osteogenesis, and therefore ameliorates osteoporosis in mice 44. Similarly, we observed that ZN27 increased the number of type H vessels in the distraction area. As angiogenesis and osteogenesis are closely coupled in the early phase of DO 27, our results suggest that in addition to the direct enhanced effects on the osteogenic process, ZN27 also improves the coupling of angiogenesis and osteogenesis via the upregulation of FAK signaling in BMSCs, which further facilitates bone formation during DO.

The limitations of this study existed in that despite the activation of the FAK-ERK1/2 signaling, other molecules and signaling pathways 43, 45 may also contribute to ZN27-induced enhancement of angiogenesis and osteogenesis. Future studies should investigate additional molecules and signaling pathways regulated by ZN27 and further verify their regulation of bone regeneration. Besides, although the mechanisms of osteogenesis during DO and other bone regeneration models are not the same because of the rhythmic mechanical stimuli in DO, FAK signaling is also important to osteogenesis in fracture 46, osteoporosis 47, etc. While we primarily focused on the effect of ZN27 on DO in the present study, the potential effect of ZN27 in other bone regeneration models could be further explored.

## Conclusion

The present study demonstrates that ZN27 improves the angiogenic capacity of HUVECs and osteogenic differentiation of BMSCs via FAK-ERK1/2 signaling. Moreover, ZN27 facilitates neovascularization and bone regeneration in DO, suggesting the therapeutic potential of ZN27 for repairing large bone defects in the mechanobiological environment during DO. Future studies are required to explore more sophisticated regulation patterns of bone regeneration by ZN27 and evaluate the efficacy of ZN27 in other medical settings.

## Supplementary Material

Supplementary figures.

## Figures and Tables

**Table 1:** Primer sequences used for qRT-PCR.

Genes	Forword primer sequence (5′-3′)	Reverse primer sequence (5′-3′)
*VEGFA (Homo sapiens)*	CAGAAGGAGGAGGGCAGAA	GTCTCGATTGGATGGCAGTAG
*CD31 (Homo sapiens)*	GGTGGAGTCTGGAGAGGACATT	GGGTGGCATTTGAGGTCATT
*GAPDH (Homo sapiens)*	TGCACCACCAACTGCTTAGC	GGCATGGACTGTGGTCATGAG
*Alp (Rattus)*	CCGCAGGATGTGAACTACT	GGTACTGACGGAAGAAGGG
*Ocn (Rattus)*	CAGACAAGTCCCACACAGCA	CCAGCAGAGTGAGCAGAGAGA
*Opn (Rattus)*	GGCCGAGGTGATAGCTT	CTCTTCATGCGGGAGGT
*Osx (Rattus)*	GGAAAAGGAGGCACAAAGAA	CAGGGGAGAGGAGTCCATT
*Runx2 (Rattus)*	ACTTCCTGTGCTCGGTGCT	GACGGTTATGGTCAAGGTGAA
*Gapdh (Rattus)*	ATGGCTACAGCAACAGGGT	TTATGGGGTCTGGGATGG

## Abbreviations

DO: Distraction osteogenesis
FAK: Focal adhesion kinase
BMSCs: Bone marrow mesenchymal stem cells
ERK1/2: Extracellular signal-regulated kinase 1/2
ZN27: ZINC40099027
HUVECs: Human umbilical vein endothelial cells
VEGFA: Vascular endothelial growth factor A
CD31: Platelet endothelial cell adhesion molecule-1
EMCN: endomucin
GAPDH: glyceraldehyde-3-phosphate dehydrogenase
ALP: Alkaline phosphatase
ARS: Alizarin red S
OCN: Osteocalcin
OPN: Osteopontin
OSX: Osterix
RUNX2: Runt-related transcription factor 2
OIM: Osteogenic differentiation induction medium
BV/TV: Bone volume/tissue volume
H&E: Hematoxylin-eosin
PSR: Picrosirius red
COL2: Collagen type II
VEGFR2: Vascular endothelial growth factor receptor 2
MSCs: Mesenchymal stem cells
