# Supplementary material for: Enhanced Bone Regeneration through Regulation of Mechanoresponsive FAK-ERK1/2 Signaling by ZINC40099027 during Distraction Osteogenesis
Source: Int J Med Sci. 2024 Jan 1;21(1):137–50. doi: 10.7150/ijms.88298 (PMC10750334; doi:10.7150/ijms.88298)
Supplement: Supplementary file 1 — Supplementary figures. [file ijmsv21p0137s1.pdf]

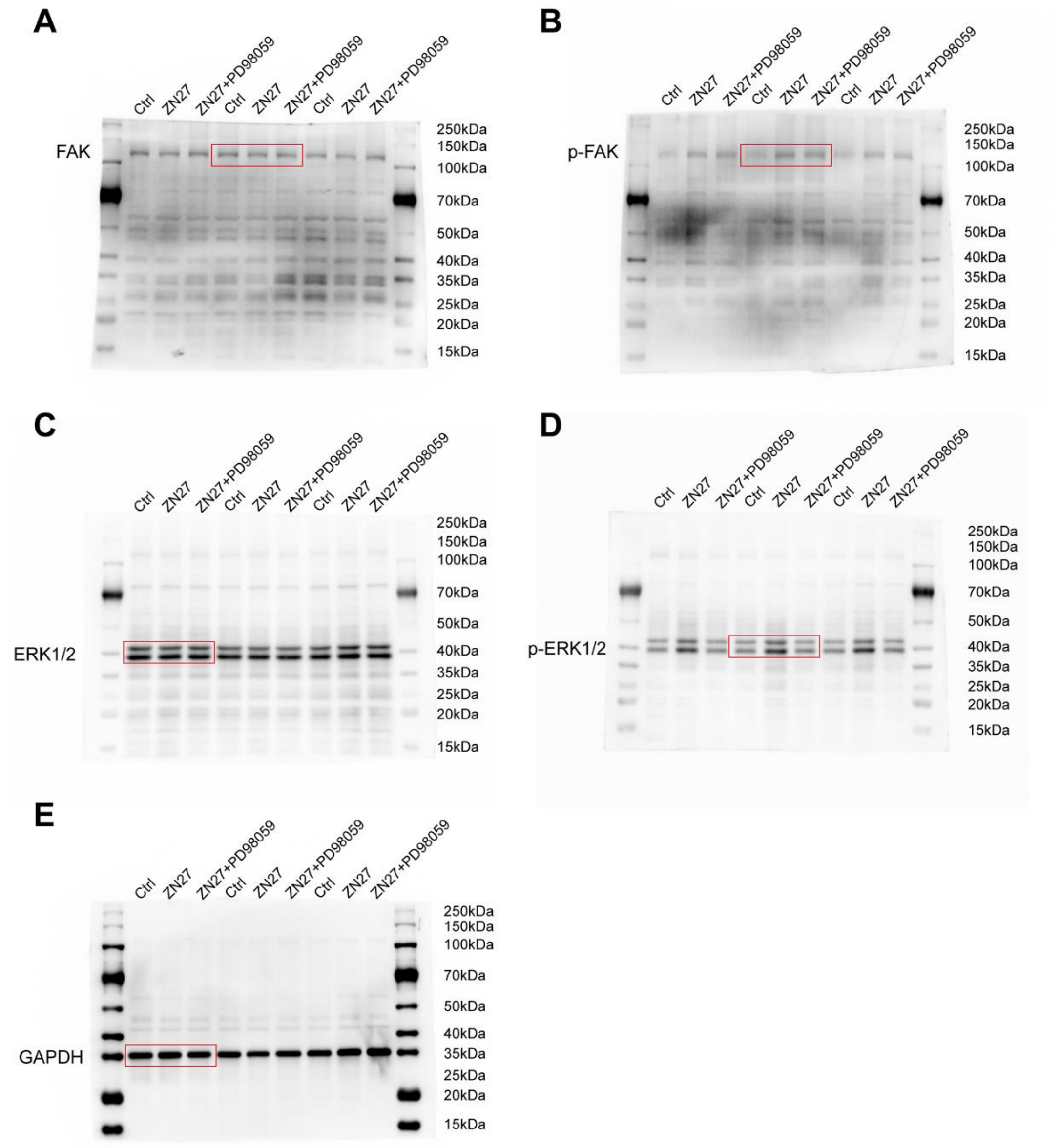

Supplementary Fig. 1 A-E: The uncropped western blot images of **Fig. 2A** and the red lines were used to indicate where they were cropped. PD98059: ERK1/2 inhibitor. Abbreviations: ZN27, ZINC40099027; FAK, focal adhesion kinase; ERK1/2, extracellular signal-regulated kinase 1/2; GAPDH, glyceraldehyde-3-phosphate dehydrogenase.

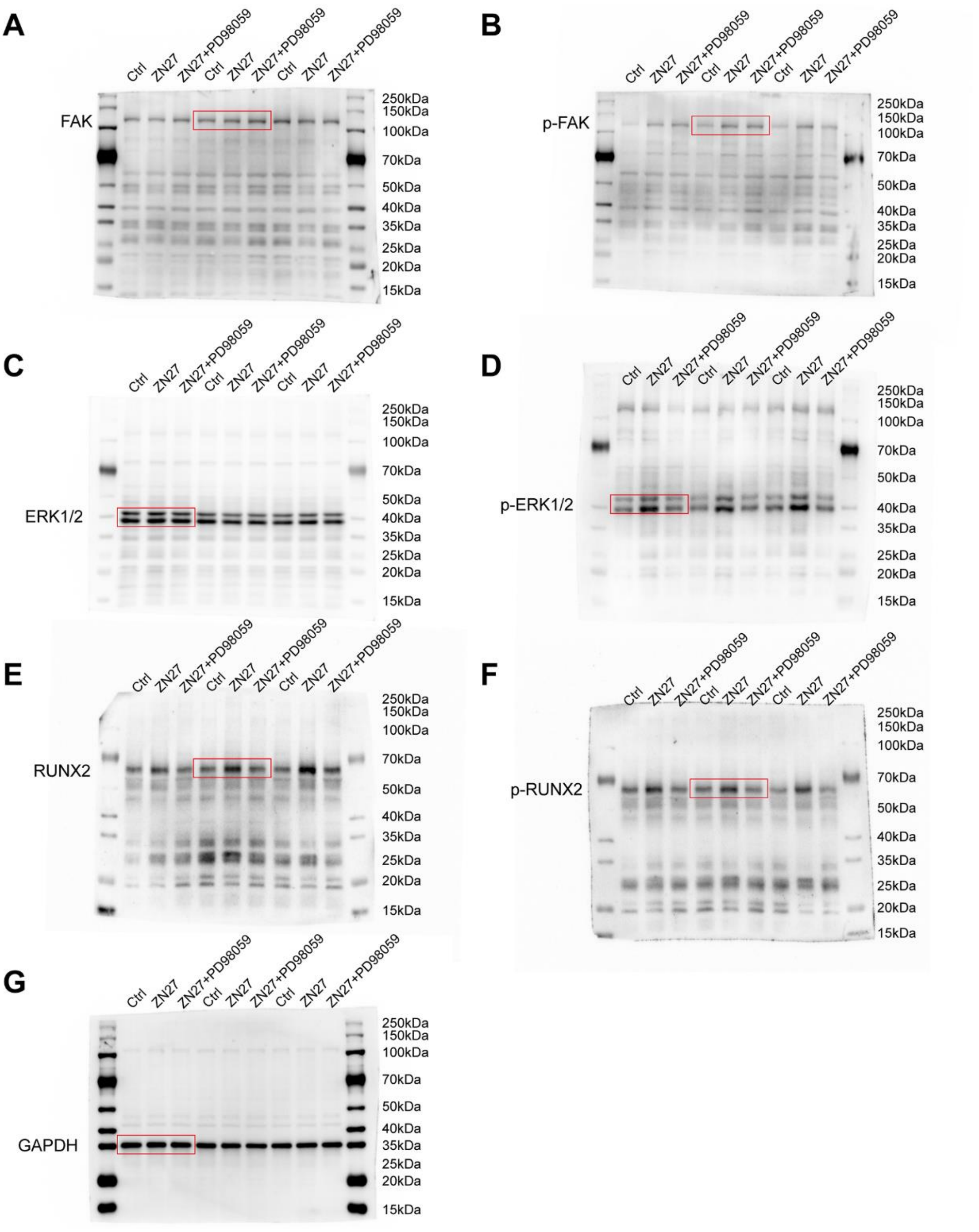

Supplementary Fig. 2 A-G: The uncropped western blot images of **Fig. 4A** and the red lines were used to indicate where they were cropped. PD98059: ERK1/2 inhibitor. Abbreviations: ZN27, ZINC40099027; FAK, focal adhesion kinase; ERK1/2, extracellular signal-regulated kinase 1/2; RUNX2, runt-related transcription factor 2; GAPDH, glyceraldehyde-3-phosphate dehydrogenase.
